# Second-Line Treatment of Non-Small Cell Lung Cancer: Clinical, Pathological, and Molecular Aspects of Nintedanib

**DOI:** 10.3389/fmed.2017.00013

**Published:** 2017-02-28

**Authors:** Luis Corrales, Amanda Nogueira, Francesco Passiglia, Angela Listi, Christian Caglevic, Marco Giallombardo, Luis Raez, Edgardo Santos, Christian Rolfo

**Affiliations:** ^1^Clinical Oncology Department, Hospital San Juan de Dios, San José, Costa Rica; ^2^Phase I – Early Clinical Trials Unit, Oncology Department, Antwerp University Hospital, Center for Oncological Research (CORE), Antwerp University, Antwerp, Belgium; ^3^Department of Surgical, Oncological and Oral Sciences, Section of Medical Oncology, University of Palermo, Palermo, Italy; ^4^Medical Oncology Department, Instituto Oncológico Fundación Arturo López Pérez, Santiago, Chile; ^5^Thoracic Oncology Program, Memorial Cancer Institute, Memorial Health Care System, Pembroke Pines, FL, USA; ^6^Oncology Department, Lynn Cancer Institute, Boca Raton, FL, USA

**Keywords:** non-small cell lung cancer, angiogenesis, target therapy, nintedanib, second-line treatment, clinical trials

## Abstract

Lung carcinoma is the leading cause of death by cancer in the world. Nowadays, most patients will experience disease progression during or after first-line chemotherapy demonstrating the need for new, effective second-line treatments. The only approved second-line therapies for patients without targetable oncogenic drivers are docetaxel, gemcitabine, pemetrexed, and erlotinib and for patients with target-specific oncogenes afatinib, osimertinib, crizotinib, alectinib, and ceritinib. In recent years, evidence on the role of antiangiogenic agents have been established as important and effective therapeutic targets in non-small cell lung cancer (NSCLC). Nintedanib is a tyrosine kinase inhibitor targeting three angiogenesis-related transmembrane receptors (vascular endothelial growth factor, fibroblast growth factor, and platelet-derived growth factor). Several preclinical and clinical studies have proven the usefulness of nintedanib as an anticancer agent for NSCLC. The most important study was the phase III LUME-Lung 1 trial, which investigated the combination of nintedanib with docetaxel for second-line treatment in advanced NSCLC patients. The significant improvement in overall survival and the manageable safety profile led to the approval of this new treatment in Europe. This review focuses on the preclinical and clinical studies with nintedanib in NSCLC.

## Introduction

Lung cancer is one of the most common malignancies in the world and is the leading cause of cancer-related deaths worldwide, accounting for 1.59 million deaths yearly. In the United States alone, an estimated 221,200 new cases of lung cancer were diagnosed in 2015, and 158,040 people will die of this disease (1–3). Non-small cell lung cancer (NSCLC) is the most frequent type of lung cancer, accounting for more than 80% of all cases, whereas small cell lung cancer represents 15–20% (4, 5). Most patients will experience disease progression during or after first-line chemotherapy, and there is a significant unmet need for new, effective second-line treatments. Currently, the only approved second-line therapies for patients who do not harbor identifiable driver oncogenes, such as epidermal growth factor receptor (*EGFR*) gene mutations or anaplastic lymphoma kinase (*ALK*) gene translocations, are docetaxel, gemcitabine, pemetrexed (limited for non-squamous NSCLC), and erlotinib (6–9).

The majority of patients with NSCLC do not achieve prolonged disease control, and the 5-year survival rate remains poor at 18.7% (1). Growing knowledge of NSCLC molecular pathobiology has led to the development of new treatments that target specific oncogenes (10) and have changed the natural history of the disease with a clear improvement of patient’s survival (11). However, it is still characterized by a significantly low survival for second-line treatment (12, 13) with a median progression-free survival (PFS) from 2 to 3 months and a median survival rarely exceeding 8 months (14). The recognition of patients harboring EGFR mutations (EGFRm) or EML4-ALK translocation and displaying tyrosine kinase inhibitors (TKI) response rates of approximately 70% account an essential treatment. With the use of molecularly targeted therapies, such as erlotinib (15), afatinib (16) for EGFRm, osimertinib (17) for EGFRm T790, and crizotinib (18), alectinib, and ceritinib (19, 20) for ALK positive (Table 1), a higher response rates and prolonged PFS have been obtained when compared to chemotherapy in the first- and second-line setting (21).

**Table 1:** **Early development of Target therapy in non-small cell lung cancer (NSCLC)**.

Drug mechanism	Reference	*N* total	Drug	Comparator	Median overall survival (OS)	Median OS regarding sequential combination of EGFR–TKI and chemotherapy	Median progression-free survival (PFS)
Tyrosine kinase inhibitors	Zhou et al. (15)	154	Erlotinib	Gemcitabine + carboplatin	22.8 versus 27.2 months^a^	29.7 versus 20.7 or 11.2 months, respectively (*p* < 0.0001)	NA
	Yang et al. (16)	631	Afatinib	Cisplatin/pemetrexed	27.3 versus 24.3 months^a^	NA	NA
				OR			
				Gemcitabine/cisplatin			
	ELCC (17)	60	Osimertinib	platinum-pemetrexed	NA	NA	19.3 months
	Noonan and Camidge (18)	343	Crizotinib	platinum-pemetrexed	^a^	NA	10.9 versus 7.0 months
	Shaw et al. (19)	130	Ceritinib	NA	NA	NA	7 months
Antiangiogenic agents	Sandler et al. (28)	878	Bevacizumab + Paclitaxel + carboplatin	Paclitaxel + carboplatin	12.3 versus 10.3 months (*p* = 0.003)	NA	6.2 versus 4.5 months (*p* < 0.001)
	Garon et al (32)	1,253	Ramucirumab + Docetaxel	Docetaxel + Placebo	10.5 versus 9.1 months (*p* = 0.023)	NA	4.5 versus 3.0 months (*p* < 0.0001)
	Blumenschein et al. (33)	52	Sorafenib	NA	6.7 months	NA	2.7 months
	Heist et al. (37)	125	Sunitinib	Sunitinib + Pemetrexed	8.0 versus 6.7 versus 10.5 months	NA	3.3 versus 3.7 versus 4.9 months
				OR			
				Pemetrexed			

*^a^not statistically significantly different*.

*NA, non-applicable*.

Antiangiogenic agents have been established as important and effective therapeutic targets in many cancers, including NSCLC. Angiogenesis is one of the hallmarks of cancer and is critical for the growth, progression, and metastasis of many solid tumor types (22–24). Mechanisms that support the formation of neovasculature include vascular endothelial growth factor (VEGF), fibroblast growth factor (FGF), and platelet-derived growth factor (PDGF) signaling pathways (22, 25–27). To date, first-line bevacizumab remains the only approved antiangiogenic treatment in the therapeutic armamentarium for advanced NSCLC. Its use is restricted to patients with tumors with a non-squamous histology (28, 29).

In the Eastern Cooperative Oncology Group, 878 patients with recurrent or advanced NSCLC were recruited and assigned to paclitaxel/carboplatin chemotherapy alone or paclitaxel/carboplatin and bevacizumab. The addition of the anti-VEGF to a standard, platinum-based doublet regimen conferred a significant prolongation in overall survival (OS), PFS, and response rate in patients with NSCLC (28) (Table 1). Also, bevacizumab administered with paclitaxel showed a median PFS longer compared to docetaxel in second-third line of treatment (30) In the AVAiL trial, patients with non-squamous NSCLC were randomized to receive cisplatin/gemcitabine with or without bevacizumab and in a similar way, the results in this trial demonstrated an improvement in PFS versus placebo (31).

Furthermore, ramucirumab, a vascular endothelial growth factor receptor-2 (VEGFR2) inhibitor, was investigated as second-line therapy with docetaxel for stage IV NSCLC. Median OS and PFS were longer in the ramucirumab arm compared with the placebo arm (32) (Table 1). Even though VEGF is the most potent angiogenic molecule, the inhibition of the VEGF pathway with TKI or monoclonal antibodies is associated with a modest survival benefit.

The multikinases inhibitor sorafenib targets VEGFR2–3, PDGFR-β, c-kit, RAF, and FLT-3. In two phase II studies, it was determined an improvement in PFS and in OS when used as a single agent with respect to placebo (33) (Table 1). Furthermore, a phase I/II trial studied the effect of sorafenib combined with carboplatin/paclitaxel and showed a median PFS of 34 weeks with a good toxicity profile (34). However, two Phase III trials, ESCAPE and NEXUS trials, were conducted to confirm the efficacy and feasibility of the combination treatment. Unfortunately, neither of the trials met their primary endpoints (35, 36).

Sunitinib, an orally selective multitargeted TKI that inhibits PDGFR, KIT, FLT-3, and VEGFR, has also been evaluated in combination with both chemotherapy and erlotinib after failure of first-line platinum-based chemotherapy. CALGB 30704 randomized patients to pemetrexed alone, sunitinib alone, or the combination of pemetrexed/sunitinib as second-line therapy for advanced NSCLC (37) (Table 1). The results demonstrated a non-statistically significant higher response rate in patients receiving pemetrexed/sunitinib and a better PFS and OS in the single agent pemetrexed arm. Also, two trials evaluated the combination of erlotinib and sunitinib, and no differences in PFS or OS were observed (38, 39).

Unfortunately, the activation of other angiogenic pathways has also developed drug resistance by the tumor. Molecules, such as FGF and PDGF, have been found upregulated in patients exhibiting acquired resistance to anti-VEGF treatment. The use of multitargeted anti-angiogenesis tyrosine kinase inhibitors (MATKIs) to achieve simultaneous inhibition of two or three angiogenic pathways has been proposed as a promising strategy for improved outcomes in NSCLC patients (40).

**Figure 1:**
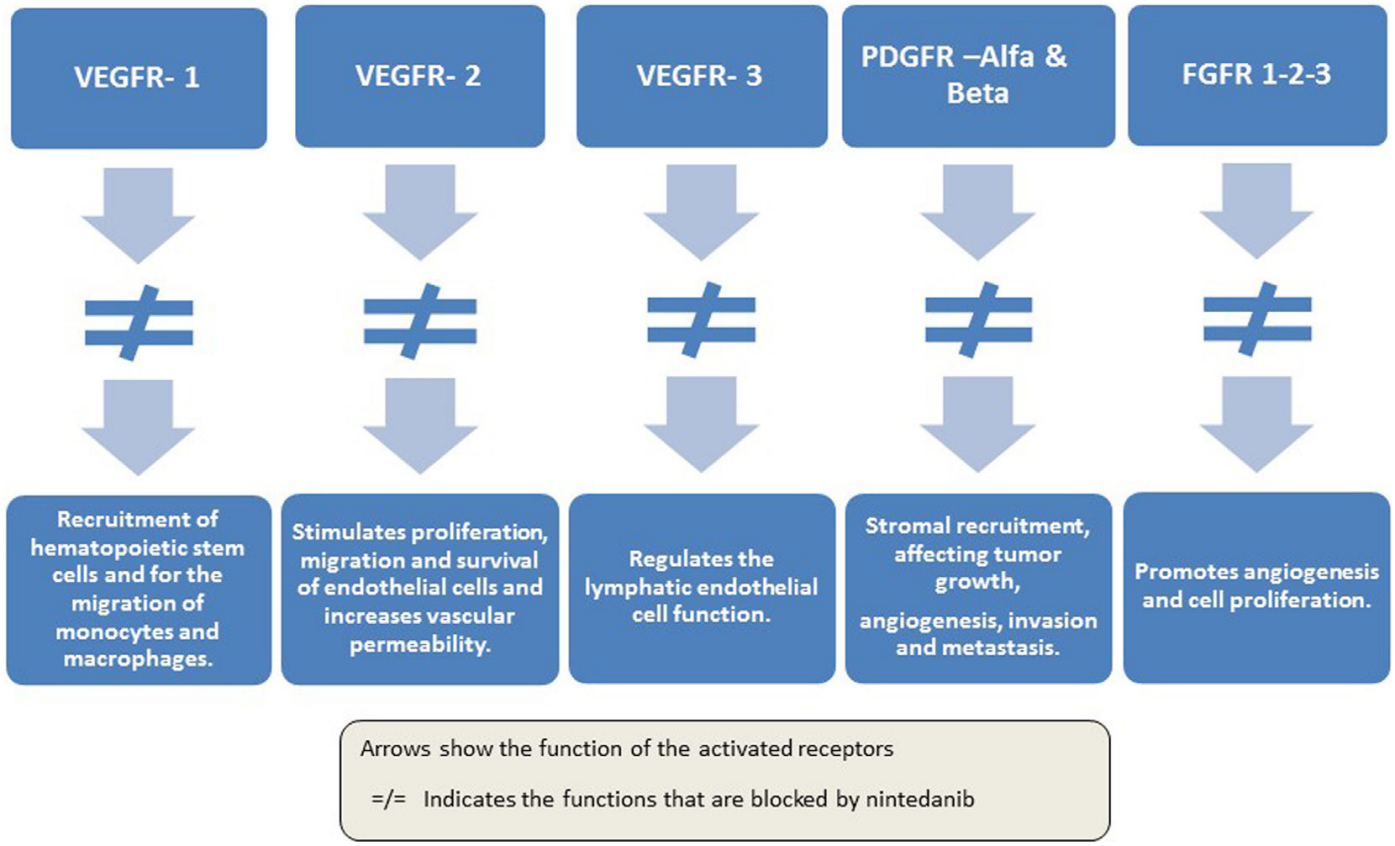
**Mechanism of action of nintedanib in lung cancer treatment**.

Nintedanib (Vargatef^®^; BIBF 1120) is a novel, potent, oral, triple angiokinase inhibitor that targets VEGF receptors 1 to 3, PDGF receptors alpha and beta, and FGF receptors 1 to 3 (41–43), as well as members of the Src family and FLT-3 (43) (Figure 1).

## Preclinical Development

Nintedanib was identified during a program for small molecule inhibitors of angiogenesis, and studies were extended to various solid tumors (43). Recent evidence shows that nintedanib is a potent endothelial cell proliferation inhibitor with a good safety profile, proven in both *in vitro* and *in vivo* studies.

This molecule, an indolinone derivative, occupies the adenosine triphosphate-binding sites in the kinase domain of pro-angiogenic receptors previously mentioned, inhibiting the downstream signaling pathways. Overall, the spectrum is fairly restricted (VEGFR-1, VEGFR-2, VEGFR-3, FGFR-1, 2, 3, PDGFR-α and β, FLT3, and SRC family member) and has shown low cross-reactivity with other human kinases (41, 43, 44). Peak plasma concentrations of nintedanib are reached 2–4 h after oral administration and have a terminal half-life of 10–15 h. Also, it is metabolized largely *via* hydrolytic cleavage by esterases; cytochrome P450 pathways have a minor role in the metabolism of the MATKI. The major route of elimination is fecal/biliary excretion (45).

*In vitro* studies showed that treatment with nintedanib induced proliferation arrest and apoptosis in endothelial cells, smooth muscle cells, and pericytes, cell types involved in angiogenesis, through the inhibition of both AKT and mitogen-activated protein kinases signaling pathways, resulting in an overexpression of the apoptosis marker cleaved caspase-3 (43).

Moreover, *in vivo* studies performed in human NSCLC xenografts have confirmed these results. One of the studies showed that at well-tolerated doses, nintedanib was highly active and demonstrated additive effects in combination with the cytotoxic drugs docetaxel or pemetrexed (42). In addition, in another study, nintedanib alone and in combination with standard chemotherapy showed a potent inhibition of proliferation and increased apoptosis of tumor cells in NSCLC xenografts that were poor responders to bevacizumab and resistant to platinum doublet chemotherapy (46). It demonstrated rapid changes in tumor vessel architecture, such as reduction of vessel permeability and perfusion, and microvessel density. Intracellularly, the inhibitory effect of nintedanib was found to be markedly sustained, with inhibition of VEGF receptor activation for at least 32 h after being treated for 1 h with nintedanib, suggesting slow receptor dissociation kinetics and sustained inhibition (43). There was no association with an increased expression of the epithelial mesenchymal transition (EMT) markers, a common mechanism of resistance to antiangiogenic therapies (46).

Another recent study evaluating the co-treatment of nintedanib with small interfering RNAs against six specific genes involved in EMT has shown that this molecule is able to do a downregulation of SYDE1 and ZEB1, and this sensitizes the cell’s response to the drug in terms of EMT reversal (47). Additionally, *in vitro* and *in vivo* studies have evaluated the toxic potential of nintedanib, showing a tolerable safety profile of this compound, excluding any severe cardiovascular, respiratory, or neurological adverse effects, as well as any mutagenic potential of nintedanib (48).

Furthermore, the combination potential of nintedanib with PD-1 antagonists was explored in an *in vivo* combination experiments in two syngeneic murine tumor models. The murine tumor cell lines CT-26 and 4T1 were injected subcutaneously into female mice and subsequently treated with RMP1-14, a murine anti PD-1, nintedanib, or RMP1-14/nintedanib. Single agent treatment of CT-26 subcutaneous tumors with RMP1-14 resulted in antitumor effect with treated to control values of 45% and nintedanib resulted in a 63%. The combination treatment group after 24 days showed a value of 34%. Additionally, the use of nintedanib in the anti PD-1 refractory model 4T1 showed a synergistic combinatorial antitumor effect. The combination of angiogenic and immune checkpoint inhibition is an attractive opportunity to improve overall response rates and efficacy based on the dual roles of angiogenic factors in blood vessel formation and immune regulation (49).

## Phase I and Phase II Clinical Trials

The tolerability of nintedanib has been studied in different kinds of neoplasm, such as ovarian cancer, NSCLC, breast cancer, colorectal cancer, urothelial carcinoma, and head and neck cancer (50). In a phase I open-label, dose-escalation trial, Doebele et al. studied the combination of this MATKI with paclitaxel and carboplatin in chemotherapy-naïve advanced NSCLC (51). Twenty-six patients enrolled and received nintedanib at the starting dose of 50 mg twice daily on days 2–21 in association with 200 mg/m^2^ paclitaxel and area under the curve 5 of carboplatin on day 1 of each 21-day cycle. Overall, 84.6% (*n* = 22) experienced a partial response or stable disease without confirmation, and 26.9% (*n* = 7) achieved a confirmed partial response. The treatment was well tolerated with liver enzyme elevations, thrombocytopenia, abdominal pain, and rash being the dose-limiting toxicities (DLT) (Table 2).

**Table 2:** **From phase I to phase III clinical trials on nintedanib**.

Clinical trial (phase)	Reference	Patient characteristics	*n*	Drug combination	*N* dose/frequency	Response *n* (%)			Stable disease	Progression	Median PFS	Median OS
						Stable disease or partial response	Partial response	Complete response				
I	Doebele et al. (51)	Chemotherapy-naïve advanced NSCLC	26	Paclitaxel + carboplatin + N	50 mg/2 id	22 (84.6)	7 (26.9)	0	15 (57.7)	NA	NA	NA

I/II	Ellis et al. (52)	Advanced NSCLC preciously treated with first-line platinum-based chemotherapy	26	Pemetrexed + N	100 mg^a^/2 id	NA	NA	1 (3.8)	13 (50)	8 (30.8)	5.4 months	NA

I	Okamoto et al. (40)	Advanced NSCLC previously treated	42	Docetaxel + N	150–200 mg/2 id	31 (73.7)	NA	NA	NA	NA	NA	NA

II	Reck et al. (54)	Stage IIIB/IV NSCLC	73	N	150 or 250 mg/2 id	43 (59)					6.9 weeks	21.9 weeks

III	LUME-Lung 1 Trial (55–57)	Stage IIIB/IV NSCLC progressing after first-line chemotherapy	1,314	Docetaxel + N	200 mg/2 id	NA	NA	NA	NA	NA	3.4 versus 2.7 months+	10.1 versus 9.1 months++

	Campos-Gomez and Campos-Gomez (61)	Advanced NSCLC progressing after one line of chemotherapy	17	Docetaxel + N	200 mg/2 id	NA	13 (81.25)	NA	3 (18.75)	NA	NA	42 months

	Garcia Montes (62)	Advanced lung adenocarcinoma who progressed to first-line treatment + bevacizumab	99	Docetaxel + N	200 mg/2 id	79 (79.6)	52 (53)	NA	26 (26.5)	16 (16.3)	NA	NA

	LUME-Lung 2 Trial (63)	Advanced non-squamous NSCLC previously treated with chemotherapy	713	Pemetrexed + N	200 mg/2 id	435 (61)	NA	NA	NA	NA	4.4 versus 3.6 months	12.2 versus 12.7 months

*N, nintedanib; n, number of patients enrolled; NA, non-applicable; +, 4.2 months when considering group of patients with adenocarcinoma; ++, 12.6 versus 10.3 months when considering group of patients with adenocarcinoma, p = 0.0359*.

*^a^Initial dose*.

In another dose-escalation phase I/II trial, 26 patients with advanced NSCLC previously treated with first-line platinum-based chemotherapy, received nintedanib in association with pemetrexed. Patients received a starting dose of nintedanib of 100 mg twice daily on days 2–21 in association with 500 mg/m^2^ of pemetrexed on day 1 of a 21-day cycle. Similar to the previous studies, the resultant maximum tolerated dose (MTD) of nintedanib was established at 200 mg twice daily. Moreover, of the enrolled patients, 1 had a complete response, 13 had stable response, and 8 patients showed progressive disease. The median PFS was approximately 5.4 months. A good safety profile was confirmed, with fatigue, anorexia, and ALT increase being the most frequent grade 3 drug-related adverse events (52) (Table 2). Moreover, in a Japanese trial, the same MTD of nintedanib (200 mg twice daily) was established and a manageable safety profile and similar efficacy results as the previous studies were found (53).

Okamoto et al. evaluated in a phase I trial, the combination of nintedanib with docetaxel in advanced NSCLC patients who had been previously treated. Forty-two patients (17 BSA < 1.5, 25 BSA ≥ 1.5) were treated. The MTD of nintedanib was 150 and 200 mg twice daily in patients with BSA less than 1.5 and BSA greater than or equal to 1.5, respectively, in combination with 75 mg/m^2^ of docetaxel. They found encouraging efficacy results, yielding a 73.7% of disease control rate. Furthermore, DLT, all grade 3 hepatic enzymes elevations, occurred in only one-third of the enrolled patients. All hepatic enzyme elevations were reversible and manageable with dose reduction or discontinuation. The main drug-related adverse events included neutropenia (95%), leukopenia (83%), fatigue (76%), alopecia (71%), decreased appetite (67%), and elevations in alanine aminotransferase and aspartate aminotransferase (64%) (40) (Table 2).

Also, a phase II double-blind study assessed the efficacy, safety, and tolerability of nintedanib in stage IIIB/IV NSCLC. The 73 patients recruited tolerated the continuous treatment and had no significant difference in efficacy between treatment arms (nindetanib 250 mg twice a day versus 150 mg twice a day). The median PFS was 6.9 weeks and the median OS was 21.9 weeks with no significant difference between the two groups; the disease control rate was 59% (54) (Table 2).

## Phase III Trials

The LUME-Lung 1 trial (NCT00805194) is a multinational, randomized, placebo-controlled phase 3 trial that assessed the efficacy and safety of the combination of nintedanib and docetaxel in patients with stage IIIB/IV NSCLC progressing after first-line chemotherapy. Patients were assigned to docetaxel 75 mg/m^2^ by intravenous infusion on day 1 in addition to nintedanib 200 mg twice daily orally or matching placebo on days 2–21, every 3 weeks. The primary endpoint was PFS, which was assessed by an independent central review, analyzed by intention to treat after 714 events in all patients. As key secondary outcome, OS was predefined and analyzed on an intention-to-treat basis in a pre-specified, stepwise, fixed-sequence order: first, in a predefined group of patients with adenocarcinoma and poor prognosis (i.e., time elapsed since start of first-line therapy of less than 9 months until randomization into the trial); second, in patients with adenocarcinoma; and finally, in all patients regardless of histology. Other secondary outcomes were investigator-assessed PFS, tumor response by central review and investigator assessment, safety, and tolerability.

The study met its primary endpoint demonstrating a statistically significant improvement in PFS that translated into a 21% reduction in risk of progression (55). The PFS according to central independent review was significantly longer in nintedanib plus docetaxel group than in docetaxel plus placebo group (median PFS 3.4 versus 2.7 months; HR 0.79; 95% CI 0.68–0.92; *p* = 0.0019), with a more pronounced benefit in patients with adenocarcinoma histology (median PFS 4.2 versus 1.5 months; HR 0.68; 95% CI 0.54–0.84; *p* = 0.0005). Also, the subset of patients with adenocarcinoma and poor prognosis had a median PFS of 4.2 months in the docetaxel plus nintedanib group versus 1.6 months in the docetaxel plus placebo group (HR 0.67; 95% CI 0.43–1.04, *p* = 0.0725) (56).

Even though, in the total population of patients there was only a trend in favoring the combination of docetaxel and nintedanib (median OS 10.1 versus 9.1 months; HR 0.94; 95% CI 0.83–1.05; *p* = 0.2720), in adenocarcinoma subgroup there was a significant difference in OS (median OS 12.6 versus 10.3 months; HR 0.83; 95% CI 0.70–0.99; *p* = 0.0359). Improvement was also observed in patients with adenocarcinoma histology and poor prognosis; the median OS was longer in the docetaxel plus nintedanib group compared with the docetaxel plus placebo group (median OS 10.9 months versus 7.9 months; HR 0.75; 95% CI 0.60–0.92; *p* = 0.0073) (56). The intent-to-treat analysis of OS in all studied patients showed a 1-month improvement that did not reach statistical significance; however, when adjusted to the sum of longest diameters of target lesions, a significant OS benefit was seen (55).

The tolerability profile was similar to that shown in phase I/II clinical trials. The adverse events that were more common in the docetaxel plus nintedanib group than the docetaxel plus placebo group were: diarrhea, increases of transaminases, nausea, decreased appetite, and vomiting, with only a 18.6% requiring dose reduction (56). Also, a study determined the impact on tumor growth over time as a treatment effect, with a specific focus on patients with poor prognosis (i.e., time of progression less than 9 months and who had progressive disease as best response to first-line treatment). The use of nintedanib and docetaxel showed a significant reduction in tumor burden and tumor growth over time compared to docetaxel in patients with adenocarcinoma histology and in the group of patients with the poorest prognosis (57) (Table 2).

Furthermore, Heigener et al. performed an analysis of adenocarcinoma population in the LUME-Lung 1 to determine if first-line treatment could influence subsequent outcomes for nintedanib and docetaxel arm. In the study, the efficacy outcomes, the OS benefit, and the frequency of adverse events were similar regardless of prior treatments with taxanes, pemetrexed, or bevacizumab (58).

Popat et al. confirmed LUME-Lung-1 findings in a meta-analysis of nine studies. They estimated a probability of 70% for nintedanib plus docetaxel being the best second-line treatment with regard to OS and PFS (59). Based on these findings, the European Medicines Agency approved in November 2014 the combination of nintedanib with docetaxel for the second-line treatment of adenocarcinoma patients (60). Furthermore, using patient-reported outcomes [i.e., 30-item European Organisation for Research and Treatment of Cancer Core Quality of Life (QoL) Questionnaire and its 13-item lung cancer-specific supplement] to complement the objective measures of efficacy and safety, this trial allowed the assessment of patients’ subjective perception of their symptom burden and health-related QoL. This analysis demonstrated that the survival benefits achieved in the LUME-Lung 1 trial were not at the expense of patients’ QoL. No significant differences in the PRO composites for cough, dyspnea, or pain were observed between the treatment groups (56).

Moreover, a cohort of NSCLC Mexican patients receiving nintedanib with docetaxel demonstrated efficacy and that was well tolerated; 81.25% had a partial response and 18.75% had stable disease (61). Also, a descriptive trial used the clinical data collection of patients with advanced lung adenocarcinoma who progressed to first-line treatment plus bevacizumab included in the compassionate-use program of nintedanib. The primary objective of the study was to describe the characteristics of the patients and their tumors, including previous therapies. The secondary objectives were to estimate the time under nintedanib treatment and the response rate and to evaluate the safety of this new treatment in daily clinical practice. From the 99 patients who were included, the objective response rate was 53%, stable disease 26.5%, disease progression 16.3%, and 4% were non-evaluable. Also, the disease control rate was 79.6%. The majority of patients had adequate tolerance, similar to the results obtained in LUME-Lung 1, mostly toxicities grades 1–2. However, the retrospective design of the study and the biased criteria of the investigator could have influenced in the overestimated responses (62).

Another phase III controlled randomized trial, LUME-Lung 2 (NCT00806819) evaluated the use of nintedanib in combination with pemetrexed (500 mg/m^2^) and compared with pemetrexed (500 mg/m^2^) plus placebo in patients with advanced, non-squamous NSCLC previously treated with chemotherapy (63). The primary endpoint was the same as LUME-Lung 1, while the secondary endpoints included OS, investigator-assessed PFS, response rate, safety, and QoL. Even though the enrollment was halted after randomizing 713 patients based on a planned futility analysis, the study met its primary endpoint. The nintedanib arm had a significant better PFS (median PFS 4.4 versus 3.6 months compared with placebo; HR 0.83; 95% CI 0.70–0.99; *p* = 0.0435); however, this difference was not translated into an OS benefit (12.2 versus 12.7 months; HR 1.03; 95% CI 0.85–1.24; *p* = 0.7921). Moreover, disease control was also significantly improved in the nintedanib arm (61 versus 53%, odds ratio 1.37, *p* = 0.039). Also, in this study, nintedanib showed a higher incidence of grade 3 increases in liver enzymes and gastrointestinal events, which resolved with dose reduction and supportive treatment (56). In contrast to other antiangiogenic agents, no grade 3/4 hypertension or hand-foot syndrome was reported (54) (Table 1).

Additionally, the association between plasma levels of VEGF, FGF, and PDGF was evaluated, both baseline and after treatment with nintedanib plus docetaxel, as well as disease control rate, PFS, and OS, among 38 patients with NSCLC. A higher percentage change reduction in PDGF after treatment was associated with a longer PFS and a higher percentage change in FGF was associated with a longer OS. Also, a higher reduction of plasma levels of FGF and PDGF was associated with better clinical outcomes (64).

Several clinical trials involving nintedanib are ongoing, including a phase III study (NCT02299141), that will evaluate the effectiveness of nintedanib in molecularly selected NSCLC patients and investigate the potential role of some genes (VEGFR1-3, PDGFR-A, PDGFR-B, and FGFR1-3) that might be involved in the regulation of mechanisms of acquired resistance to antiangiogenic agents. Results are expected by June 2017.

## Conclusion

Nintedanib might be a good treatment option that fulfils the unmet need for effective, well-tolerated treatment options in advanced NSCLC and alleviate the disease burden for a broad selection of patients. The significant improvement in PFS in the overall population and the subgroup of patients with adenocarcinoma observed with the addition of nintedanib to cytotoxic drug therapy represents an attractive second-line treatment option. Moreover, the safety profile of this MATKI is manageable, giving this new treatment option great potential as an emerging combination for the management of NSCLC.

## Author Contributions

All authors contributed equally to this paper. All authors agreed to be accountable for the content of the work.

## Conflict of Interest Statement

The authors declare that the research was conducted in the absence of any commercial or financial relationships that could be construed as a potential conflict of interest.
